# Improvements in quality of life of patients with multiple sclerosis receiving alemtuzumab in clinical practice: the LEMVIDA study

**DOI:** 10.1186/s41687-024-00822-9

**Published:** 2024-12-18

**Authors:** José Eustasio Meca-Lallana, Sara Eichau, Bonaventura Casanova, Elena Álvarez Rodríguez, Antonio Pato, Mireia Forner, Baldo Toledo

**Affiliations:** 1 Multiple Sclerosis CSUR, Neurology Department, Hospital Clínico Universitario Virgen de la Arrixaca, Murcia, Spain; 2 Clinical Neuroimmunology and Multiple Sclerosis Department, UCAM, Universidad Católica San Antonio, Murcia, Spain; 3 Neuroimmunology Unit, Hospital Virgen Macarena, Sevilla, Spain; 4 Hospital Universitari i Politècnic La Fe, Valencia, Spain; 5 Hospital Álvaro Cunqueiro, Vigo, Spain; 6 Hospital Povisa, Vigo, Spain; 7 Sanofi, Barcelona, Spain

**Keywords:** Alemtuzumab, Clinical outcome assessments, Multiple sclerosis, Quality of life, Relapsing-remitting multiple sclerosis

## Abstract

**Background:**

Alemtuzumab is a humanized monoclonal antibody approved for the treatment of relapsing-remitting multiple sclerosis (RRMS). Its efficacy and safety have been widely demonstrated in clinical trials, but experience from real-world cohorts is also needed to support its clinical use. Quality of life (QoL) outcomes are an important complement to the clinical benefits of treatment, offering a patient-centered perspective on how the drug contributes to general well-being. In this line we aimed to evaluate the QoL of patients treated with alemtuzumab in clinical practice.

**Methods:**

This prospective 3-year multicenter study was carried out in adult patients diagnosed with RRMS who had started alemtuzumab according to clinical practice within 8 weeks before inclusion. The primary endpoint was the change in QoL over three years of treatment with alemtuzumab using the 29-item Multiple Sclerosis Impact Scale (MSIS-29). Secondary endpoints included changes from baseline in the 21-item Modified Fatigue Impact Scale (MFIS-21), Beck Depression Inventory (BDI-II), Symbol Digit Modalities Test (SDMT, oral version) and Work Productivity. Disability worsening was also assessed based on the Expanded Disability Status Scale (EDSS), along with the annualized relapse rate (ARR) and radiological activity.

**Results:**

A cohort of 165 patients was analyzed (mean age 38.6 years, mean disease duration 8.5 years, mean EDSS score 3.3). MSIS-29 physical domain scores decreased significantly from baseline by a mean of 7.2 ± 1.8 points at year 1, 6.4 ± 2.2 at year 2 and 5.6 ± 2.3 at year 3 (*p* < 0.05 in all cases). Similarly, MSIS-29 psychological domain scores decreased significantly by a mean of 7.9 ± 2.4 points at year 1, 12.8 ± 2.9 at year 2 and 13.2 ± 3.0 at year 3 (*p* < 0.05 in all cases). Significant reductions from baseline were also evidenced in MFIS-21 and BDI-II scores, while SDMT scores remained unchanged. During the 3 years on alemtuzumab, the ARR was 0.15, representing an 83% reduction from the 2 years before initiation. At 3 years, 81.5% of patients were free from radiological activity and 87% were free from disability worsening.

**Conclusions:**

These results indicate early and substantial improvements in patients’ perception of their QoL and functioning with alemtuzumab that were sustained over three years.

**Supplementary Information:**

The online version contains supplementary material available at 10.1186/s41687-024-00822-9.

## Background

Multiple sclerosis (MS) is a chronic and disabling neurological disease that affects approximately 2.8 million people worldwide, with a 2020 global prevalence rate estimated of 35.9 cases per 100,000 people, varying between geographical regions and ethnic groups [1]. Impaired mobility caused by MS and symptoms such as fatigue, pain, cognitive dysfunction, depression and spasticity are known to impair patient quality of life (QoL), affecting the physical and psychological domains of functioning and well-being [2–4]. In recent years, QoL has become a valuable outcome in MS research and clinical care to gain a broader understanding of the impact of MS on patients’ lives and, most importantly, the success of treatment from their perspective and the level of care they require [5, 6]. Assessing QoL can inform neurologists about how patients are experiencing the treatment, which is a good step in promoting shared decision-making [7].

Alemtuzumab is an anti-CD52 monoclonal antibody approved for the treatment of relapsing-remitting multiple sclerosis (RRMS), that has been shown in clinical trials to reduce clinical relapses, disability progression, magnetic resonance imaging (MRI) lesion activity and brain volume loss, with a well-known safety and tolerability profile [8–13]. Real-world studies have replicated these findings and provided clinicians with a more realistic picture of the benefits and risks of the drug [14–22].

Many alemtuzumab-treated patients from phase 3 CARE-MS clinical trials have reported improvements in physical and psychological aspects of QoL [23] that were sustained over 5 and 6 years [24, 25]. In clinical practice, few studies have examined the impact of alemtuzumab on patient QoL, but the results are consistent. The interim analysis of the PROMiS study [26] showed significant and clinically meaningful improvements in QoL over 8 months after alemtuzumab. In turn, the PRO-ACT study [27], although not primarily designed to assess QoL, also demonstrated improvements in physical and psychological functioning at 24 months, and the INFUSE-MS study [28] reported a positive physical and psychological effect of the drug.

Inflammatory and radiological activity and physical disability only partially reflect the impact of disease. The possibility of having a highly effective drug that also improves patient wellbeing is of particular interest, and in recognition of this value, we focused on capturing the patient’s perspective with alemtuzumab, which has practically not been explored to date.

The aim of this study was to evaluate the QoL of patients treated with alemtuzumab in clinical practice. As fatigue, depression and cognitive impairment have a detrimental effect on patient function and are closely related to QoL, we also investigated these manifestations to better understand and interpret the data obtained on this complex and multidimensional construct. Although in an exploratory manner, we also evaluated the experience of patients treated with alemtuzumab on productivity across employed work and household chores.

## Methods

### Study design and patient population

The LEMVIDA study was a 3-year prospective observational study conducted in accordance with the Declaration of Helsinki and national regulations.

Between October 2016 and September 2018, a total of 167 adults diagnosed with relapsing-remitting multiple sclerosis (RRMS) who had initiated alemtuzumab according to clinical practice within the 8 weeks before inclusion were recruited from 40 Spanish centers, regardless of prior therapy exposure. Eligible patients had to be able to complete the patient-reported outcome (PRO) measures MSIS-29 (29-item Multiple Sclerosis Impact Scale), MFIS-21 (21-item Modified Fatigue Impact Scale) and BDI-II (Beck Depression Inventory), and the performance outcome (PerfO) measure SDMT (Symbol Digit Modalities Test).

Alemtuzumab infusions were administered according to the Summary of Product Characteristics (SmPC). During treatment, neurologists adhered to the Risk Management Program in Europe for alemtuzumab to ensure early detection of adverse events and compliance with monitoring requirements. Any treatment decision during the study was made at the physician’s discretion.

The study protocol was approved by the Ethics Committee of the Hospital Clínico San Carlos (Madrid, Spain), and all patients gave written informed consent to participate.

### Assessments

Patients were followed-up for up to 36 months, with regular clinical visits every 6 months.

Demographic and clinical characteristics were collected, including the number of relapses in the previous two years and during follow-up (defined as acute/subacute episodes of new/increasing neurological dysfunction present for at least 24 h, followed by full/partial recovery in the absence of fever or infection), MRI activity (gadolinium [Gd] - enhancing or new/enlarging T2 lesions in the last pre-alemtuzumab MRI, and at month 12, 24 and 36, when available), and the level of disability using the Expanded Disability Status Scale (EDSS).

Patient QoL was assessed using the MSIS-29 scale [29]. MSIS-29 evaluates the physical and psychological impact of MS on a scale from 0 to 100, which lower scores indicating better QoL. MSIS-29 scores can also be categorized from 0 to 19 as ‘no problems’, 20–39 as ‘few problems’, 40–59 as ‘moderate problems’, 60–79 as ‘quite a few problems’ and 80–100 as ‘extreme problems’. Levels of fatigue, depression and cognitive status were determined using the MFIS-21 scale [30], the BDI-II inventory [31], and the oral version of the SDMT [32], respectively. MFIS-21 is a 21-item scale that rates the impact of fatigue on function using a 5-point scale (never to always). MFIS-21 higher scores indicate greater impact of fatigue. The maximum score is 84, and a cut-off point < 38 has been proposed as no significant fatigue [33]. BDI-II is a 21-item self-report inventory widely used to measure the severity of depression. The range of scores is 0 to 63, with higher scores indicating a greater severity of depression. BDI-II scores are classified from 0 to 13 as ‘minimal depression’, from 14 to 19 as ‘mild depression’, from 20 to 28 as ‘moderate depression’ and from 29 to 63 as ‘severe depression’ [31]. The SDMT is the most sensitive instrument of cognitive function in MS that measures patient attention, concentration and speed information [34]. The final score is the correct number of substitutions in 90 seconds (range between 0 and 110), and the cut-off of ≤ 49 correct substitutions is used to identify participants with cognitive problems [32].

The impact of MS on productivity was measured using the health-related productivity questionnaire (HRPQ).

Safety of alemtuzumab was assessed by collecting all adverse events (AEs) (serious or non-serious) that occurred during the study, related or suspected to be related to alemtuzumab and resulting in permanent discontinuation of the drug.

### Statistical analysis

The primary endpoint was the change in MSIS-29 scores over 3 years of treatment with alemtuzumab. Descriptive statistics of the MSIS-29 scores in each physical and psychological dimensions are presented, including mean ± standard deviation (SD). Changes from baseline in scores over 3 years were determined using the Hotelling Trace coefficient, and the Least Significant Difference (LSD) method was used to calculate the differences between two means.

Secondary endpoint analyses included changes from baseline in the EDSS, and total MFIS-21, BDI-II and SDMT scores. The Hotelling Trace coefficient was also used to determine the change in scores over 3 years, along with the LSD method to test differences between means.

The annualized relapse rate (ARR) was calculated using the person-years method, which divides the number of all observed relapses among all patients by the total follow-up period of the study cohort and the 95% confidence interval (CI). Post hoc analyses were performed to explore NEDA-3 (no evidence of disease activity), defined as “no protocol-defined relapses, no Gd-enhancing lesions or new/enlarging T2 lesions, and no 6-month confirmed disability worsening (CDW)”. CDW was defined as increases in the EDSS score of ≥ 1.5 point from a baseline EDSS score of 0, ≥ 1.0 point from a baseline EDSS score of 1 to ≤ 5.5, or ≥ 0.5 point from a baseline EDSS score of > 5.5 for at least 6 months. NEDA-3 was calculated yearly and cumulative. Patients who progressed in the last 6 months of the planned 36 months of follow-up were excluded from the analysis of the annual 24–36 months and the cumulative 1–3 years, because they did not have an additional follow-up assessment to confirm progression. Overall and annual incidence of reported AEs is presented.

Statistical significance was defined as *p* < 0.05, and analyses were performed with SPSS Statistics version 28.0.

## Results

### Patients characteristics

Of the 167 enrolled patients, two were excluded as screening failures. The mean age was 38.6 ± 9.1 years, and 67.9% were female. Patients had a mean time since diagnosis of 8.5 ± 6.0 years, a mean EDSS score of 3.3 ± 1.7, and a mean number of relapses in the previous 2 years of 1.8 ± 1.3. Most patients (93.3%) had received prior disease-modifying treatments (DMTs) (Table 1).

Nine patients did not receive the second course of alemtuzumab. Two patients received a third course 12 months after the second course, and 7 patients received a third course approximately 2 years after the second course. Forty patients (24.2%) discontinued alemtuzumab and withdrew from the study. The common reasons for discontinuation were loss to follow-up (*n* = 10, 25%), insufficient effectiveness (*n* = 8, 20%), change of medication (*n* = 8, 20%), and patient decision (*n* = 4, 10%).

**Table 1:** Baseline patient characteristics (N = 165)

Patient characteristics	Value
**Age** (years), mean ± SD	38.6 ± 9.1
**Sex**, n (%)	
Male	53 (32.1)
Female	112 (67.9)
**Time from diagnosis** (years), mean ± SD	8.5 ± 6.0
**EDSS**	
Mean ± SD	3.3 ± 1.7
Median (IQR)	3.0 (2.0–4.0)
**Patients with relapses in the past 2 years**, n (%)	141 (85.4)
Relapses in the previous 2 years, mean ± SD	2.1 ± 1.1
**Patients with Gd-enhancing lesions**, n (%)^a^	72 (47.7)
Number of Gd-enhancing lesions, mean ± SD	5.1 ± 6.8
**Prior DMT use**, n (%)	154 (93.3)
Number of previous DMTs, mean ± SD	2.3 ± 0.9
Fingolimod, n (%)	66 (42.9)
Natalizumab, n (%)	30 (19.5)
Platform DMTs, n (%)^b^	53 (34.4)
Other therapies, n (%)^c^	4 (2.5)

DMT, disease modifying therapy; EDSS, Expanded Disability Status Scale; Gd, gadolinium; IQR, interquartile range; SD, standard deviation. ^a^Missing data, *n* = 14; ^b^Platform therapy included: interferon beta 1a/1b, *n* = 14; glatiramer acetate, *n* = 12; dimethyl-fumarate, *n* = 17; teriflunomide, *n* = 10; ^c^Other therapies included: mitoxantrone, *n* = 1; azathioprine, *n* = 1; rituximab, *n* = 2; ocrelizumab, *n* = 1

### Quality of life

**Fig. 1:**
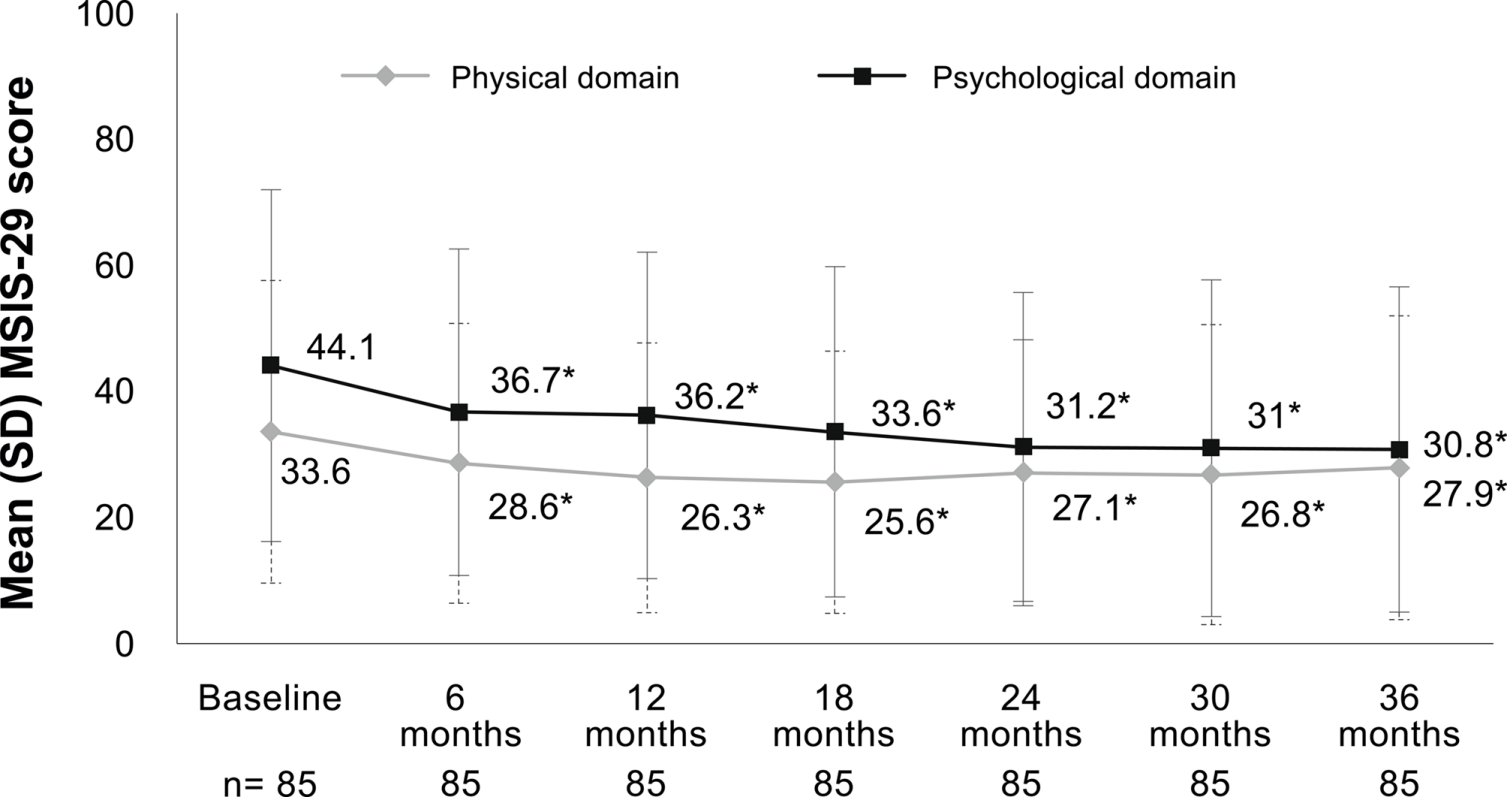
Changes in multiple sclerosis impact scale over 3 years of treatment with alemtuzumab. Mean differences between baseline and follow-up times were all significant (*p < 0.05 vs. baseline). Significant change over 36 months in the physical domain (*N* = 85; *p* = 0.007) and psychological domain (*N* = 85; *p* = 0.002), by GLM repeated measures-Hotelling trace. MSIS-29 categories: 0–19 = no problems; 20–39 = few problems; 40–59 = moderate problems; 60–79 = quite a few problems; 80–100 = extreme problems. GLM, general linear model; MSIS-29, 29-item Multiple Sclerosis Impact Scale; SD, standard deviation

The MSIS-29 scores showed significant changes in physical domain over 36 months (*p* = 0.007) and a mean decrease versus baseline of 7.2 ± 1.8 points at year 1 (*p* < 0.001), 6.4 ± 2.2 points at year 2 (*p* = 0.006) and 5.6 ± 2.3 points at year 3 (*p* = 0.017) (Fig. 1). MSIS-29 psychological domain scores decreased significantly over 3 years (*p* = 0.002), with a mean decrease versus baseline of 7.9 ± 2.4 points at year 1 (*p* = 0.002), 12.8 ± 2.9 points at year 2 (*p* < 0.001) and 13.2 ± 3.0 points at year 3 (*p* < 0.001) (Fig. 1).

### Fatigue, depression and cognitive function

**Fig. 2:**
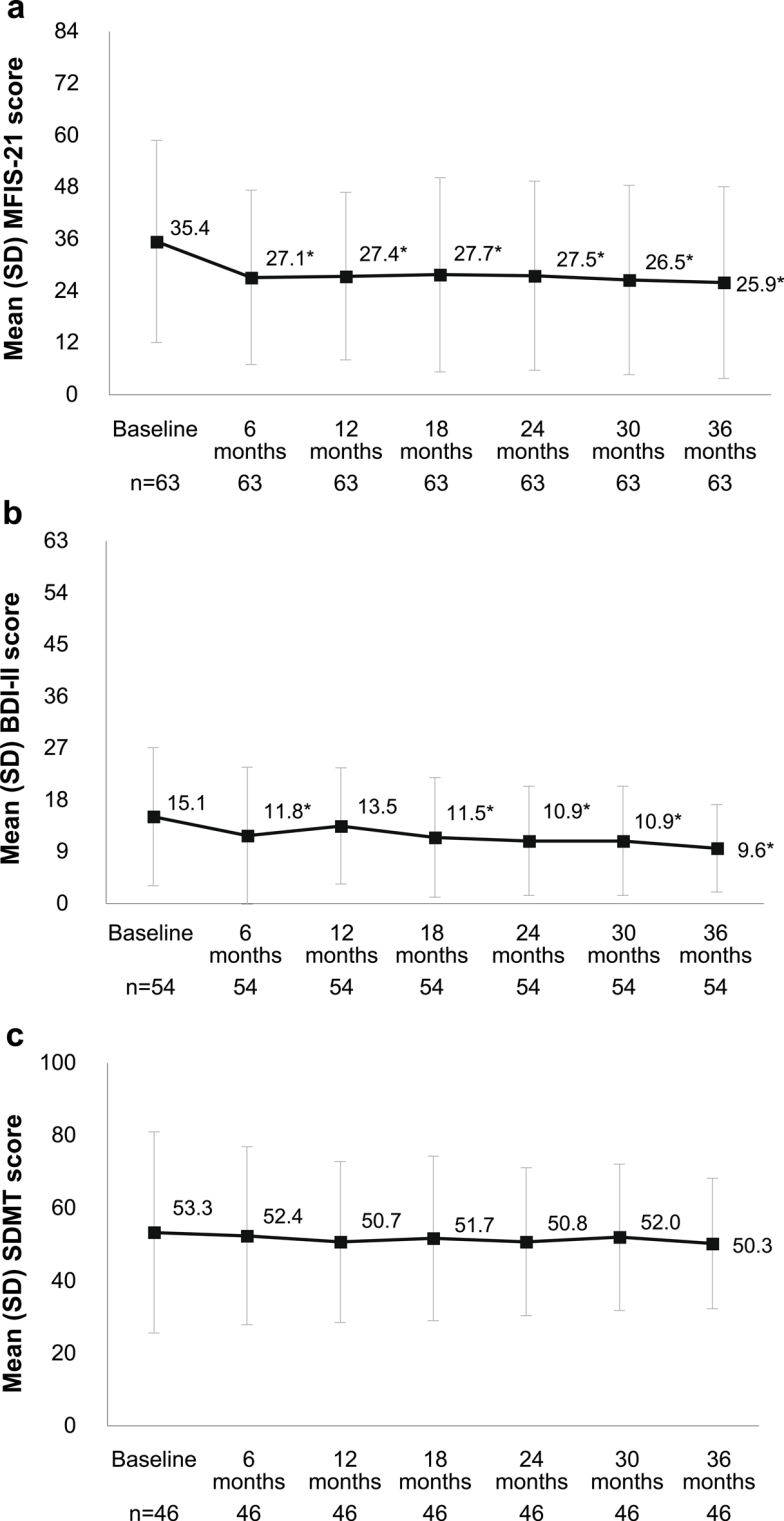
Changes in levels of fatigue (**a**), depression (**b**) and cognitive function (**c**) over 3 years of treatment with alemtuzumab. **a** Mean differences between baseline and follow-up times were all significant (*p < 0.05 vs. baseline). Significant change over 36 months (*N* = 63; *p* < 0.040) by GLM repeated measures-Hotelling trace. MFIS-21 cut-off point: <38, absence of fatigue. Higher scores indicate more levels of fatigue. **b** Mean differences between baseline and follow-up times were all significant except between baseline and month 12 (*p < 0.05 vs. baseline). Significant change over 36 months (*N* = 54; *p* < 0.009) by GLM repeated measures-Hotelling trace coefficient. BDI-II categories: 0–13 = minimal depression; 14–19 = mild depression; 20–28 = moderate depression; 29–63 = severe depression. **c** No significant change over 36 months (*N* = 46; *p* = 0.940) by GLM repeated measures-Hotelling trace coefficient. SDMT cut-off point: ≤49 successes indicate problems with information processing speed. BDI-II, Beck Depression Inventory-II; GLM, general linear model; MFIS-21, 21-item Modified Fatigue Impact Scale; SDMT, Symbol Digit Modality Test; SD, standard deviation

The MFIS-21 scores decreased significantly from baseline to month 36 (*p* = 0.002), with a mean change of 7.9 ± 2.2 points at year 1 (*p* < 0.001), 7.9 ± 2.7 points at year 2 (*p* = 0.005) and 9.5 ± 3.0 points at year 3 (*p* = 0.003) (Fig. 2a). Likewise, the BDI-II scores decreased significantly from baseline (*p* = 0.009), with a mean change of 1.1 ± 1.0 points at year 1, 4.2 ± 1.2 points at year 2 and 5.5 ± 1.3 points at year 3 (Fig. 2b). The baseline SDMT scores of 53.3 ± 27.7 remained without significant changes from 1 to 3 years after starting alemtuzumab (Fig. 2c).

### Work productivity

Overall, the percentage of patients who had to stop working due to MS or treatment decreased from 31.6% at baseline to 14.3% at year 3 (Supplementary Table 1). The mean number of hours missed due to MS or the treatment decreased from 35.6 at baseline to 15.2 at year 3, while the median number of hours planned for housework increased from 5 at baseline to 9.5 at year 3.

### Effectiveness

**Fig. 3:**
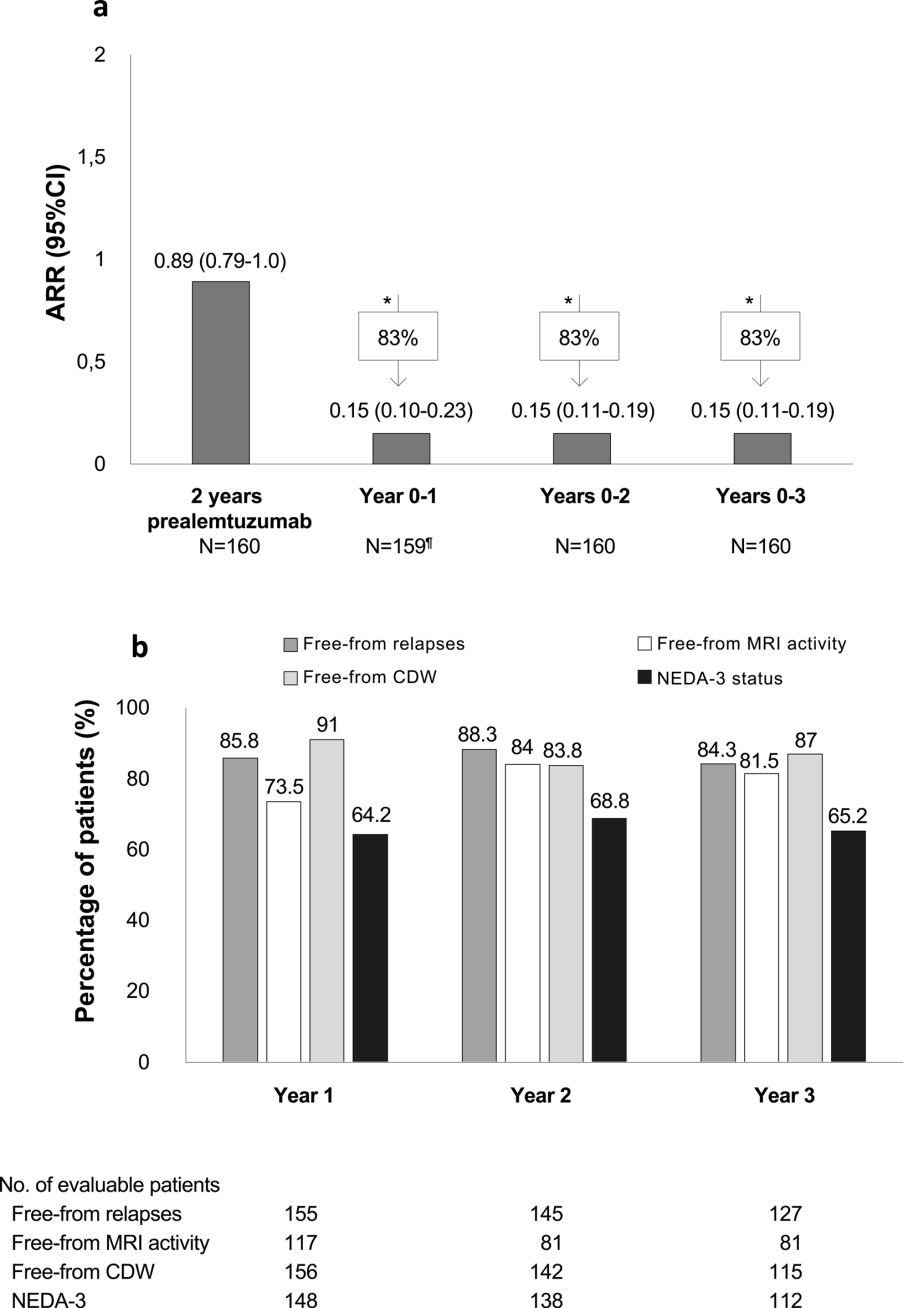
Annualized relapse rate during alemtuzumab treatment (**a**) and percentage of patients who achieved NEDA-3 status and were free from relapses, MRI activity and disability worsening (**b**). *p < 0.05 vs. ARR at baseline. ^¶^ One patient with no data at 6 months and year 1. CDW, confirmed disability worsening; CI, confidence interval; MRI, magnetic resonance imaging; NEDA, no evidence of disease activity

The ARR decreased significantly by 83% from 0.89 (95% CI: 0.79-1.0) at baseline to 0.15 (95% CI: 0.10–0.23) at 1 year of alemtuzumab, and it remained at 0.15 from two to three years (Fig. 3a). At year 3 of treatment, 84.3% of patients were free from relapses, 81.5% were free from radiological activity, 87% were free from disability worsening, and 65.2% achieved the NEDA-3 status (Fig. 3b). Cumulatively over years 1–3, 50.8% of patients achieved NEDA-3 status.

### Safety

A total of 148 patients (89.7%) experienced at least one AE related to alemtuzumab, mainly skin rash (*n* = 53, 32.1%), headache (*n* = 52, 31.5%), pyrexia (*n* = 33, 20%), lymphopenia (*n* = 26, 15.8%) and pruritus (*n* = 21, 12.7%). All these AEs were graded as mild to moderate. The annual incidence of AEs was highest after the first course of treatment (46.6%) and subsequently declined to 34.8% after the second course of treatment, and to 16.7% after the third course. The annual incidence of AEs in patients with a delayed third course of alemtuzumab was 2.2%.

Thirty-two patients reported 55 SAEs during the study, 15 of which were considered to be related to alemtuzumab. Related SAEs were infusion-associated reactions reported in two patients and the following in one patient (0.6%) each: lymphopenia, thrombocytopenia, thyroid disorder, pyrexia, immune reconstitution inflammatory syndrome, influenza infection, urinary tract infection, hemophagocytic lymphohistiocytosis, elevated transaminases, sensitive MS relapse, maculopapular rash, pruritic rash, and papilloma excision. The case of hemophagocytic lymphohistiocytosis was fatal. During treatment with alemtuzumab, 19 women became pregnant, and 6 of them had 7 abortions, none of which were judged to be related to the drug.

## Discussion

LEMVIDA was an observational and prospective study that describes patient-reported experience with alemtuzumab on QoL, fatigue, depression, cognitive function and work productivity, using standardized clinical outcome assessments (COAs). This is one of the few studies to comprehensively analyze the effect of alemtuzumab from the patient perspective in a real-life cohort.

We found that in patients with a relapsing-remitting clinical course, mean age of 38 years, disease duration of 8 years, mild disability (EDSS score of 3.0) and mostly pretreated at the beginning of treatment, alemtuzumab significantly improved the psychological and physical aspects of QoL over three years.

The mean change observed in the MSIS-29 psychological domain was greater than that reported in the physical domain, probably because the patients perceived themselves to be more affected psychologically than physically when they entered the study, as indicated by the higher baseline scores. In fact, the baseline BDI-II and MFIS-21 scores showed a patient population with mild depressive symptoms but no fatigue, respectively.

The improvement observed in the mean score of MSIS-29 psychological domain represents a change in category from moderate problems to few problems. This is consistent with the effect of alemtuzumab we found on patients’ depressive symptoms, which improved significantly from mild to minimal. However, the low level of fatigue at baseline was essentially maintained, as was the baseline SDMT score of 55, indicating no cognitive decline at the start of treatment. There is evidence that depression, fatigue and specific aspects for cognitive decline are associated with QoL [2, 3]. Therefore, the differences observed in the physical and psychological impact of MS in our patients may lie in the degree of the impact (the scale does not capture the mildest levels) or in the nature of the impact (not all patients are affected in the same way in the same areas). In fact, the available evidence from studies of alemtuzumab using the MSIS-29 scale indicates a greater absolute change in the psychological domain compared to the physical domain [26–28].

In our cohort, the mean score reduction in the psychological domain of the MSIS-29 reached the minimal important change (MIC) of 5.5 [35]. However, the mean score change in the physical domain did not reach the 7.5-point change accepted as the threshold for identifying a clinically significant change in the physical impact of MS [36]. This may be related to the low levels of disability that our patients presented at baseline (mean EDSS score of 3.0), but also to the longitudinal stabilization of EDSS we found. Two studies have shown a weaker relationship between the MSIS-29 physical scale and EDSS in patients with low EDSS scores (0–4), attributed to an intrinsic defect in the construction of the EDSS. In this range, the assessment of EDSS is more focused on neurological rather than on functional impairment, which probably has little effect on the patient perception of disease impact [36, 37]. In addition, the MSIS-29 may be limited in evaluating changes related to DMTs in patients with minimal disability [38], but our results did show significant improvements in both the psychological and physical domains of the scale after treatment with alemtuzumab. This apparent discrepancy highlights the need for further research to assess the impact of alemtuzumab on the QoL of patients with MS who are minimally disabled.

In spite of this, we consider that our findings of improved QoL with alemtuzumab are meaningful. Firstly, because they are consistent with the existing evidence from the alemtuzumab phase 3 trials [23–25] and observational studies [26–28], regardless of the instrument used and the population analyzed; and secondly, because they provide a good starting point for further evaluation of the drug from the patient perspective in other populations. Indeed, we believe that the significant reduction in the low levels of fatigue and the maintenance of good cognitive performance on SDMT with alemtuzumab should not be interpreted as a poor response to the drug, but rather as an indication of a positive effect on these symptoms. These findings are also noteworthy because fatigue, depression and cognitive function under alemtuzumab have been little explored in the real-world setting, though the few data available are consistent and align with our results. The LEM-COG study [39] and the studies by Hvid et al. [40] and Riepl et al. [41] were consistent in showing a positive effect (i.e., stability and improvement) of alemtuzumab on cognitive status, depressive symptoms and fatigue.

Recently, it has been shown that patients who deteriorated in employment status after two years due to MS had more self-reported cognitive problems, more depressive symptoms, higher fatigue and higher physical disability at baseline [42]. It would have been interesting to investigate whether the stabilization of cognitive status and the positive changes in QoL, fatigue and depression with alemtuzumab were related to the good employment outcomes we observed.

The added value that QoL outcomes bring to the benefit of treatment must be balanced against the clinical outcomes. In this study, two courses of alemtuzumab led to significant disease control over the course of the study, with most patients not requiring retreatment. The ARR improved substantially within the first year of alemtuzumab and remained low for three years, with more than 80% of patients remaining relapse-free during treatment. The EDSS scores remained stable, and between 87% and 91% of patients did not show disability worsening. Radiological disease activity decreased considerably from the first year of treatment, with more than 70% of patients being free from MRI lesions during treatment.

These findings are supported by data from clinical trials [9, 12, 43] and other observational studies in similar populations [17, 18]. The good annual NEDA-3 status in the first year of treatment with alemtuzumab was also observed at two and three years of follow-up. When this result was analyzed cumulatively, the percentage decreased because it is difficult to maintain the three criteria in the long term [44, 45]. Nevertheless, we can affirm that 51% of patients receiving alemtuzumab in this cohort achieved and maintained the NEDA-3 status during the first three years of treatment, which supports previously reported values [16–18].

Safety findings were consistent with the current safety profile of alemtuzumab, and no new safety concerns were identified. The most frequent AEs were rash, headache and pyrexia, which is in line with what is expected in the SmPC [46]. During the study, 81% of patients had no serious AEs (SAEs). In those patients who did experience a SAE, the common autoimmune thyroid disorder associated with alemtuzumab was reported in only one patient. Very low incidence rates were also observed for lymphopenia, (0.6%), urinary infection (0.6%) and immune reconstitution inflammatory syndrome (0.6%).

Potential criticisms of this study could be the lack of a comparator group, and the fact that patients were not sufficiently affected by their symptoms of fatigue, depression and cognitive impairment at inclusion to see a clinically meaningful change. Strengths include that the analysis of QoL, fatigue, depression and cognitive function was performed using only patients with values for the instruments at each timepoint, thus avoiding any bias in the estimation of change due to missing data over time. Moreover, we used a disease-specific instrument to measure QoL, which is likely to be more sensitive to changes in patients’ perceptions. The study also provides a comprehensive overview of COAs and clinical data in RRMS patients treated with alemtuzumab and followed-up in 40 Spanish centers, increasing the generalizability of the results.

## Conclusions

This study shows that patients’ perception of their quality of life and functioning improved early and significantly with alemtuzumab. These benefits were sustained over three years and are consistent with the clinical benefits in terms of relapses, radiological activity and disability that we have also observed with the drug.

## Electronic supplementary material

Below is the link to the electronic supplementary material.

Supplementary Material 1

## Data Availability

The datasets used and/or analysed during the current study are available from the corresponding author on reasonable request.

## Abbreviations

AE: Adverse event
ARR: Annualized relapse rate
BDI-II: Beck Depression Inventory
COAs: Clinical outcome assessments
EDSS: Expanded Disability Status Scale
HRQ: Health-Related Productivity Questionnaire
MFIS-21: 21-item Modified Fatigue Impact Scale
MRI: Magnetic resonance imaging
MS: Multiple sclerosis
MSIS-29: 29-item Multiple Sclerosis Impact Scale
NEDA: No evidence of disease activity
PerfO: Performance outcome
PROMs: Patient-reported outcome measures
QoL: Quality of life
RRMS: Relapsing-remitting multiple sclerosis
SDMT: Symbol Digit Modalities Test
