# Supplementary material for: Improvements in quality of life of patients with multiple sclerosis receiving alemtuzumab in clinical practice: the LEMVIDA study
Source: J Patient Rep Outcomes. 2024 Dec 18;8:148. doi: 10.1186/s41687-024-00822-9 (PMC11655941; doi:10.1186/s41687-024-00822-9)
Supplement: Supplementary file 1 — Supplementary Material 1 [file 41687_2024_822_MOESM1_ESM.docx]

**Supplementary Table 1.** **Work performance/daily activity, based on HRPQ-MS v.2**

| **Question** | **Baseline** | **Month 6** | **Month 12** | **Month 18** | **Month 24** | **Month 30** | **Month 36** |
| --- | --- | --- | --- | --- | --- | --- | --- |
| **Scheduled hours of work outside home during last week**, mean±SD | 29.4 (16.1)  (n=65) | 31.3 (13.5)  (n=62) | 31.6 (14.4.)  (n=54) | 32.3 (12.4)  (n=48) | 33.7 (12.5)  (n=50) | 33.7 (12.0)  (n=33) | 32.3 (12.3)  (n=40) |
| **Stopped working due to MS or treatment,** n (%) | 18 (31.6)  (n=57) | 5 (8.6)  (n=58) | 5 (10.2)  (n=49) | 4 (8.3)  (n=48) | 7 (14.0)  (n=50) | 3 (8.3)  (n=36) | 6 (14.3)  (n=42) |
| **No. of hours missed due to MS or treatment**, mean±SD | 35.6 (27.0)  (n=17) | 17.2 (18.8)  (n=5) | 26.5 (18.4)  (n=4) | 3.9 (4.2)  (n=4) | 11.5 (14.6)  (n=6) | 22.5 (24.7)  (n=2) | 15.2 (16.8)  (n=5) |
| **Impact of MS on productivity**, median (IQR) | 15 (0.3 - 50.0)  (n=52) | 0.0 (0.0-20.0)  (n=53) | 2.0 (0.0 - 20.0)  (n=44) | 5.0 (0.0 - 21.3)  (n=46) | 12.5 (0.0 - 30.0)  (n=42) | 0.0 (0.0 - 10.0)  (n=32) | 0.0 (0.0 - 30.0)  (n=39) |
| **No. of hours of housework planned,** median (IQR) | 5.0 (1.0 - 13.0)  (n=149) | 7.0 (2.0 - 14.0)  (n=127) | 6.0 (2.0 - 14.0)  (n=126) | 8.0 (2.8 - 12.0)  (n=118) | 8.0 (3.0 - 14.0)  (n=111) | 7.0 (2.0 - 20.0)  (n=105) | 9.5 (3.0 - 15.8)  (n=88) |
| **Stopped housework due to MS or treatment,** n (%) | 52 (44.1)  (n=118) | 38 (36.2)  (n=105) | 40 (37.4)  (n=107) | 28 (28.3)  (n=99) | 22 (24.7)  (n=89) | 21 (24.1)  (n=87) | 26 (31.3)  (n=83) |
| **No. of hours of housework missed due to MS or treatment**, median (IQR) | 5.0 (3.0 - 9.8)  (n=48) | 4.0 (3.0 - 7.0)  (n=32) | 3.0 (2.0 - 7.0)  (n=34) | 4.0 (2.0 - 8.3)  (n=26) | 5.0 (2.8 - 7.3)  (n=22) | 6.0 (2.0 - 10.0)  (n=18) | 5.0 (3.0 - 11.5)  (n=24) |
| **Impact of MS on housework**, median (IQR) | 50.0 (10.0 - 60.0)  (n=114) | 20.0 (0.0 - 50.0)  (n=100) | 20.0 (0.0 - 50.0)  (n=97) | 20.0 (0.0 - 50.0)  (n=89) | 20.0 (0.0 - 50.0)  (n=85) | 20.0 (0.0 - 50.0)  (n=82) | 20.0 (0.0 - 50.0)  (n=80) |

Health-Related Productivity Questionnaire (HRPQ); IQR, interquartile range; MS, multiple sclerosis; SD, standard deviation
